# Effect of Blue Light Intensity During Spreading on the Aroma of Green Tea

**DOI:** 10.3390/foods14081308

**Published:** 2025-04-09

**Authors:** Youyue He, Yan Tang, Shiyue Song, Lailong Li, Shaoshuai An, Guoming Zhou, Jing Zhu, Song Li, Yue Yin, Anburaj Jeyaraj, Chunju Peng, Xinghui Li, Guanghui Zeng

**Affiliations:** 1 Wenzhou Key Laboratory of Early Sprouting Tea Breeding, Wenzhou Vocational College of Science and Technology (Wenzhou Academy of Agricultural Sciences), Wenzhou 325006, China; 2022204005@stu.njau.edu.cn (Y.H.); tangyan@njfu.edu.cn (Y.T.); chunjupeng@163.com (C.P.); 2 College of Horticulture, Nanjing Agricultural University, Nanjing 210095, China; 2024104079@stu.njau.edu.cn (S.S.); geneanbu1986@gmail.com (A.J.); lxh@njau.edu.cn (X.L.); 3 China Huaneng Group Co., Ltd., No. 6, FuXingMenNei St, Xicheng District, Beijing 100031, China; lailong_li@chng.com.cn (L.L.); shsh_an@jsgs.chng.com.cn (S.A.); nuptandy@163.com (G.Z.); zjing21cn@sina.com (J.Z.); 4 Nanjing Agro-Tech Extension and Service Center, Nanjing 210029, China; njlisong@126.com (S.L.); 15851075264@163.com (Y.Y.)

**Keywords:** green tea, blue-light intensity, sensory quality, volatile compounds, spreading, key odorants, Headspace solid-phase microextraction-Gas chromatography-mass spectrometry (HS-SPME-GC-MS)

## Abstract

Spreading is the key process for ensuring green tea quality. However, the effect of blue light intensity conditions on the formation of green tea aroma and the evolution of key volatile compounds has not been assessed to date. Four tea samples treated with different light conditions (blue light intensities) were used to investigate the effect of spreading treatment on changes in the composition and content of volatile compounds. Volatile compounds in green tea samples were detected using headspace-solid phase microextraction and gas chromatography-mass spectrometry under different light conditions. Orthogonal partial least squares discriminant analysis (OPLS-DA) and relative odor activity value (rOAV) analyses were then applied to clarify the best blue light condition for forming aroma and associated compounds. The 116 volatile compounds were detected in the green tea samples, of which alcohols were the most abundant. The findings demonstrated that MBL (middle-intensity blue light; 150 μmol/(m^2^∙s)) treatment was the most effective condition for developing an intense and persistent fruity and floral scent compared to HBL (high-intensity blue light; 300 μmol/(m^2^∙s)) and LBL (low-intensity blue light; 75 μmol/(m^2^∙s)). This study underscores how blue light intensity conditions shape green tea aromas and offers operational insights. It also provides a theoretical basis for controlling light conditions in the process of green tea spreading

## 1. Introduction

Teas are typically categorized into three main groups according to their fermentation levels: non-fermented (green and white teas), semi-fermented (oolong), and fully fermented (black and pu-erh teas) [1]. In recent years, the health benefits of green tea have led to its rising popularity, especially in East Asian countries [2]. The production of green tea generally consists of four stages: spreading, pan firing, rolling, and drying [3]. During green tea production, spreading is intended to lower the moisture content of tea leaves and significantly affects their physical and chemical characteristics, such as tannins, polyphenols, theine, amino acids, vitamins, and minerals [4]. In general, spreading is a preliminary and indispensable craft closely related to the formation of the desired sensory quality of made tea [5].

The quality of tea’s flavor is determined by aroma and taste, which are dependent on volatile and non-volatile compounds [6]. Aroma plays a vital role in determining the quality of tea, which includes a variety of aroma compounds, such as esters, alcohols, acids, ketones, and terpenes [7]. Although green tea has 260 volatile compounds, not all of them have an impact on the tea’s aroma [8]. In Laoshan green tea, a total of 50 aroma compounds were detected, of which 24 were key contributors to OAV > 1, indicating that these compounds contributed significantly to the overall aroma of tea [9]. Furthermore, in Enshi Yulu green tea, 134 volatile compounds were found using HS-SPME and GC-MS; however, only 25 of these were considered significant because their rOAV was more than 1 [10]. Thus, the overall fragrance of green tea is determined by only a few significant compounds.

Light is a major environmental factor that influences the production of metabolites in plants. Stimulating with particular monochromatic or polychromatic light can boost the biosynthesis of various secondary metabolites [11]. During the withering/spreading process of tea in the postharvest period, light can improve the quality of tea. Using blue and red light during the withering process significantly decreased the grassy taste in tea leaves; blue light boosted the floral scent, while red light boosted the fruity scent [12]. Red-light withering, as opposed to natural withering, can greatly enhance the taste quality of summer–autumn black tea by lessening its bitterness and astringency. Exposure to red light during withering can greatly enhance glycosidase activity in the later stages, thereby boosting the aroma quality of black tea in later processing [13]. Exposure to red light primarily interferes with the biosynthesis of secondary metabolites and pathways related to amino acid synthesis and metabolism. It enhances the accumulation of amino acids and theaflavins, affecting the chemical foundation of black tea quality [14]. Under red and blue light treatments, the tea retained its natural form, dark green color, fresh aroma, and mellow taste; the tea exposed to green light had a pure aroma and a smooth taste, while the red light added a floral aroma [15]. Using warm-colored light during the spreading process greatly enhanced the overall quality of green tea, especially with yellow light [16]. Nonetheless, with our present knowledge, the impact of changes in blue light intensity on green tea volatiles remains unclear, necessitating additional research.

This study used DARK spreading as a control and designed blue light intensity treatments to uncover how different blue light intensities affects the aroma and volatile metabolites of green tea. Through the quantification and analysis of volatile compounds, the study elucidated the influence of various blue light intensities on green tea aroma and found the best conditions for spreading. Key differential volatile compounds resulting from varying blue light intensity treatments were identified using significant difference, variables important in the projection (VIP), and rOAV analysis. The findings are expected to offer theoretical backing for technological advancements and quality enhancement of green tea. The in-depth study of blue light intensity provided a theoretical basis and technical support for the regulation and quality control of green tea aroma under different blue light intensities during the spreading process.

## 2. Materials and Methods

### 2.1. Plant Materials and Treatments

**Figure 1:**
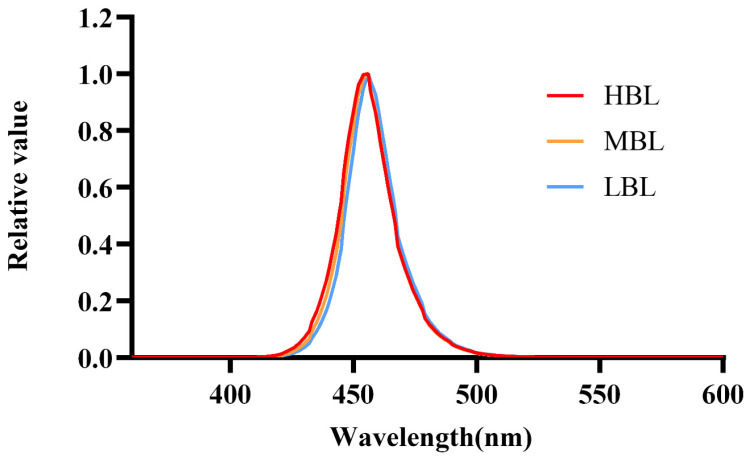
Spectral photon distribution (SPD) of blue light equipment.

The tea plant variety, “Fudingdabai” (*Camellia sinensis*), is suitable for the manufacture of green tea, which was selected for the experiments. Fresh tea leaves, consisting of one bud and two leaves, were picked from Jiangsu Tea Expo Park in Nanjing, China. First, the collected fresh leaves were evenly spread on the withering equipment with three different blue light intensity-emitting diodes (LED, 420–500 nm) [low-intensity blue light (LBL, 75 μmol/(m^2^∙s)), middle-intensity blue light (MBL, 150 μmol/(m^2^∙s)); high-intensity blue light (HBL, 300 μmol/(m^2^∙s))] (Figure 1) and the control group (DARK) was treated in the dark. All groups were subjected to spreading for 8 h under an indoor temperature of 22–26 °C. Next, they were fixed at 200 °C for 2 min. Ultimately, the tea leaves underwent drying at 80 °C for 120 min for further analysis.

### 2.2. Sensory Analysis of Green Tea Aroma

Sensory analysis referred to the method of Zhu et al. [17], with a slight adjustment. The tea infusion was prepared by brewing 3 g of green tea with 150 mL of boiling pure water. Following this, a sensory test was conducted on the tea infusion, and each tea sample was assigned a three-digit code. The samples were randomly given to 6 (3 men and 3 women) panelists who were trained specially for a quantitative descriptive analysis (QDA) of intensity values and aroma descriptors after brewing for 5 min. Six sensory attributes of tea samples were determined as follows: baked, green/grass, sweet, floral, fruity, and pungent. A scale of 0 to 10 was used to measure the intensities of the aroma attributes; the higher the scores, the stronger the intensities. Intensity levels were categorized as follows: 0 for none or not perceptible, 3 for weak, 5 for moderate, 7 for high, and 10 for extremely high. Each sample underwent three evaluations per panelist, with results presented as the mean.

### 2.3. Green Tea Aroma Extraction by HS-SPME

Volatile matter was extracted using HS-SPME and analyzed via GC-MS (Trace 1310/TSQ 9000, Thermo Fisher Scientific, Waltham, MA, USA). Microextraction fiber PDMS/DVB (65 μm) was used to adsorb volatile substances. The 1.50 g tea sample was placed in a 20-mL headspace vial, with 200 ng of ethyl caprate (Merck company, Darmstadt, Germany) serving as the internal standard, and then maintained at 80 °C to adsorb the volatile components for 40 min. Subsequently, the fiber head was desorbed at 250 °C for 5 min.

### 2.4. GC-MS Analysis of Volatile Composition

On a DB-5MS capillary column (30 mm × 0.25 mm × 0.22 μm, Agilent, Santa Clara, CA, USA), separation was carried out at 50 °C and held for 3 min, ramped to 100 °C at 10 °C/min, then ramped to 200 °C at 4 °C/min and held for 1 min, and ramped to 280 °C at 16 °C/min and maintained for 7 min. High-purity helium (>99.999%) was used at a flow rate of 1 mL/min. The MS was operated in electron impact ionization mode with an electron energy of 70 eV, ion source temperature of 230 °C, and a full scan range of 29–400 *m*/*z*. Volatiles were identified using the National Institute of Standards and Technology (NIST) mass spectral database and retention indices calculated from N-alkanes (C3–C25). The relative quantification of these compounds was determined by the peak area ratios of the quantitation ions of the internal standard, after normalizing according to the total peak area. Three replicates for the HS-SPME-GC-MS analysis were performed for each treatment.

### 2.5. rOAV Value Analysis

To determine the influence of aroma compounds, rOAV is commonly utilized, and compounds with an rOAV > 1 are regarded as key contributors to the aroma [18]. The rOAV [19] represents the proportion of volatile compound concentration in a component compared to the odor threshold. The odor threshold (OT) has been reviewed by Leffingwell and Associates Threshold Value Database (https://www.leffingwell.com/, (accessed on 12 December 2024)) and other literature. The method of Xie et al. [20] was used to calculate the rOAV value of the compound.

### 2.6. Statistical Analysis

One-way ANOVA was used to determine the significance of differences with SPSS version 26.0, and Duncan’s test identified significant differences (*p* < 0.05). SIMCA 14.1 (Umetrics, Umeå, Sweden) was used to perform the OPLS-DA. Radar plot and heat map were obtained by Origin Pro (v2024, Originlab Corporation, Northampton, MA, USA).

## 3. Results

### 3.1. Aroma Profiles

**Figure 2:**
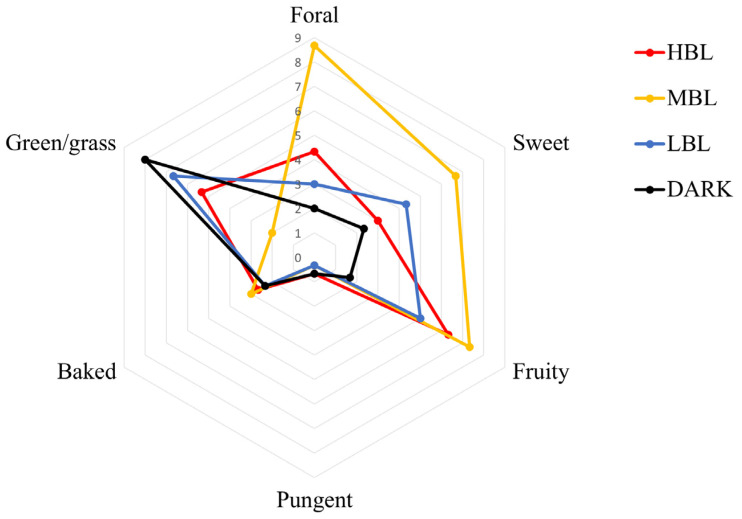
The radar of sensory aroma attributes profiles in green teas under different light conditions.

Tea infusions commonly have baked, green, sweet, floral, fruity, and pungent smells but their intensity varies. The aroma of green tea infusions was analyzed through sensory evaluations (Figure 2). The evaluations of the four tea samples revealed that the HBL treatment exhibited strong fruity aromas, moderate floral and baked scents, and a faint pungent odor. In contrast, the MBL treatment was characterized by strong sweet, floral, and fruity aromas, along with a faint green/grass odor. LBL treatment exhibited a strong green/grass and fruity scent, along with moderate floral and sweet aromas. In the DARK treatment, tea infusion displayed a strong green/grass scent and faint floral, sweet, and fruity aromas.

### 3.2. Identification and Quantification of the Aroma Compounds in Green Tea Samples by HS-SPME/GC-MS

**Figure 3:**
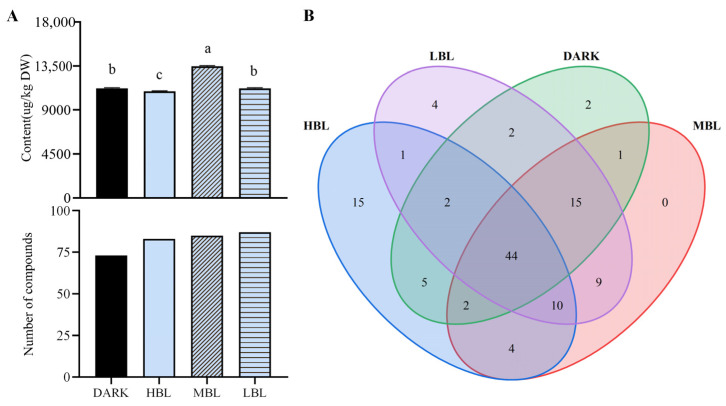
Total amount and quantity of volatile substances. (**A**) Overall aroma concentration (upper, μg/kg tea leaves relatives to internal standard (ethyl caprate) and the number of compounds (lower) conducted in the tea leaves spread in different light conditions; (**B**) Venn diagram of aroma compounds of green tea under different light conditions. Different letters in the same column indicate a significant difference between light conditions (*p* < 0.05).

GC-MS analysis showed significant differences in the volatile components of samples under various light conditions. The concentration of aroma and the number of volatile compounds differed significantly across various treatments. The total volatile concentration in MBL (13,463.39 ± 70.28 μg/kg) treatment was notably higher than in the DARK treatment (11,223.39 ± 58.37 μg/kg). In contrast, HBL (10,921.94 ± 47.52 μg/kg) treatment had a reduced aroma concentration, while LBL (11,234.43 ± 51.91 μg/kg) treatment matched that of the DARK treatment (Figure 3A). All green tea samples treated with blue light [HBL (83), MBL (85), and LBL (87)] had a greater number of aroma compounds detected than those treated with DARK treatment (73). Although the total aroma compound concentration of LBL treatment did not have statistically significant differences with the Dark treatment, the number of aroma compounds was the highest (Figure 3A). A Venn diagram (Figure 3B) was used to illustrate the logical connections among the four treatments, focusing on the key aroma compound. There were 44 shared aroma compounds found across the 4 treatments. Only 15, 4, and 2 aroma compounds were identified in HBL, LBL, and DARK, respectively. In short, the Venn diagram showed significant differences in the constitution of aroma compounds among treatments.

**Figure 4:**
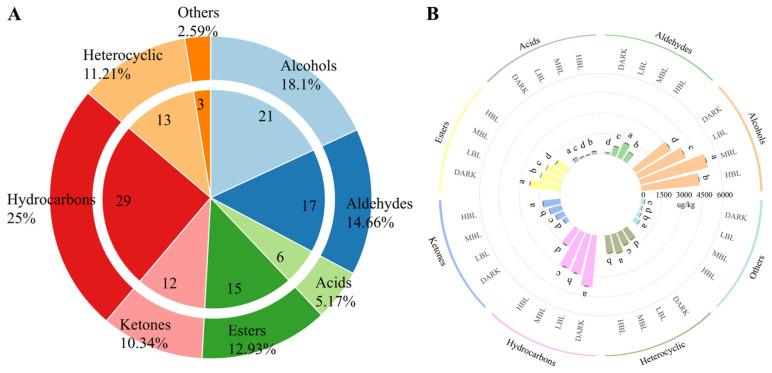
Classification and content of volatile substances. (**A**) Category and contents of compounds in green tea samples under different light conditions. (**B**) Content comparison of different categories of volatile compounds. Different letters indicated significance difference (*p* < 0.05).

All 4 green tea samples shared 116 volatile compounds, as detailed in Table 1. Additionally, these compounds were sorted into 7 different chemical classes according to their chemical structure, including alcohols, aldehydes, ketones, esters, acids, hydrocarbons, heterocyclic compounds, and others, as depicted in Figure 4A. An analysis of the distribution of volatile compounds in each category showed that in green teas under different light treatments, the largest groups were hydrocarbons (29) and alcohols (21), followed by aldehydes (17), esters (15), heterocyclic compounds (13), and ketones (12). Conversely, acids (6) and others (3) had the fewest subclasses. Alcohols and hydrocarbons were the primary groups of aroma compounds, making up nearly 43% of the volatile compounds in green tea.

Figure 4B shows the distribution of various volatile compound categories under four different light irradiation treatments. In particular, the MBL treatment had a notably higher alcohol content than the other treatments. Geraniol and phenylethyl alcohol levels were highest in the MBL treatment, attaining 1613.60 ± 3.57 μg/kg and 1003.42 ± 0.9 μg/kg, respectively. Linalool levels peaked at 415.93 ± 0.85 μg/kg in the MBL treatment and dropped to 175.16 ± 4.36 μg/kg in the DARK treatment. In the DARK treatment, esters and acids had the highest contents, measuring 2275.36 ± 21.95 μg/kg and 298.14 ± 2.72 μg/kg, respectively. Aldehydes, which play a crucial role in tea aroma, were found in concentrations ranging from 1217.62 ± 14.12 μg/kg in the MBL treatment to 148.88 ± 0.75 μg/kg in the DARK treatment. In the HBL treatment, the levels of ketones and other compounds were the highest, measuring 1301.51 ± 7.73 μg/kg and 444.96 ± 1.73 μg/kg, respectively. Heterocyclic compounds showed a marked difference in distribution, varying from 1465.24 ± 2.82 μg/kg under MBL treatment to 990.09 ± 4.26 μg/kg under DARK treatment. The highest levels of hydrocarbon levels were found in the DARK (3780.83 ± 28.06 μg/kg), followed by LBL (3039.02 ± 32.82 μg/kg), MBL (2917.03 ± 29.38 μg/kg), and HBL (1503.98 ± 23.40 μg/kg).

### 3.3. Multivariate Statistical Analysis of Volatile Compounds

**Figure 5:**
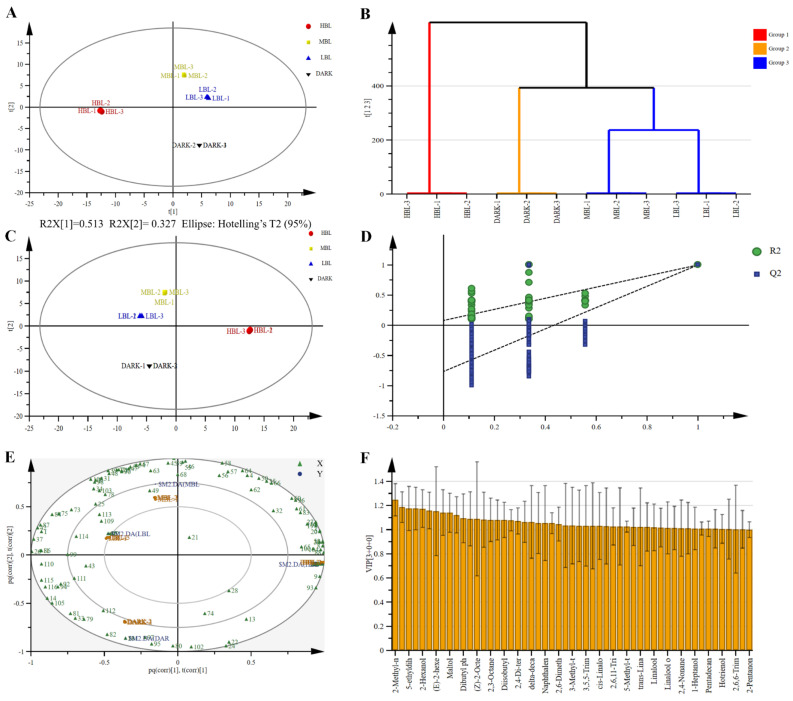
Multivariate statistical analysis of volatile compounds identified under different light conditions. (**A**) Principal component analysis; (**B**) Hierarchical cluster analysis; (**C**) Orthogonal partial least squares discriminant analysis. R2X = 0.995, R2Y = 0.999, Q2 = 0.999. (**D**) Hypothesis testing of the OPLS-DA model. (**E**) Orthogonal partial least squares discriminant analysis loading plot for volatile compounds. X represents 116 volatile compounds, and Y represents 4 light treatments. The numbers in the figure correspond to the serial number of substances in Table S1. (**F**) Orthogonal partial least squares discriminant analysis VIP plot with volatile compounds in green tea treated with different light conditions. Yellow bars represent volatile compounds with VIP > 1.

Principal component analysis (PCA) is one of the most widely used data dimensionality reduction algorithms. The PCA score plot (Figure 5A) of the four green tea samples reveals unique characteristics of the samples with good reproducibility. The first two principal components, which together account for 84% of the variation, explaining 51.3% and 32.7% of the total variance, respectively. The use of hierarchical clustering analysis (HCA) revealed the similarities and differences between the groups. According to the results, MBL and LBL were in the same cluster, while HBL and DARK were in a different cluster (Figure 5B). The clustering revealed sensory similarities and differences in volatile composition related to sensory characteristics between four green tea samples exposed to different lighting, closely matching previous PCA study results.

OPLS-DA is a multivariate statistical analysis method, which is mainly used for classification and feature selection. The purpose of OPLS-DA is to distinguish samples from different groups and identify key variables that affect group classification. To explore the differences in primary compounds of samples under various lighting conditions, OPLS-DA models were constructed based on PCA and HCA analysis. According to the findings of the PCA and HCA analysis, in this study, the score plot showed a distinct separation between various sample groups, with the MBL and LBL samples clustering closer together (Figure 5C).

R2X and R2Y denote the interpretation rates for the X and Y matrices in the constructed model, while Q2 signifies the model’s predictive capability. All three metrics are nearly 1, suggesting the model’s stability and reliability. During the analysis, the fit index for the independent variable (R2X) was found to be 0.992, and for the dependent variable (R2Y), it was 0.999. Furthermore, the model prediction index (Q2) was determined to be 0.993, suggesting a strong fit with excellent predictive accuracy. To examine the model’s robustness more thoroughly, 200 permutation tests were conducted. The results showed that the OPLS-DA model has no overfitting, and model validation was effective (Figure 5D). The differences in green tea compounds according to the light condition were more clearly shown in the OPLS-DA loading plot (Figure 5E). Specifically, the levels of compounds, like maltol, 4-vinylphenol, limonene, dibutyl phthalate, 2,6,11-trimethyl-dodecane, and 2,3-octanedione, were notably greater in the MBL and LBL samples compared to the other treatments. Conversely, the levels of compounds, like hexanal, 4-methyl- tridecane, indole, hexanoic acid ethyl ester, and 3-methyl-butanoic acid, were notably elevated in the HBL samples. Compounds, such as 6-methyl-tridecane, 4-methyl-tridecane, 3-methyl-tridecane, and 2-ethyl-1-hexanol, were present in much higher concentrations in the DARK samples.

VIP was employed to pinpoint the key volatile components that distinguished the two groups. Components with VIP scores greater than 1 were typically deemed crucial for aroma quality. To explore key aroma compounds, VIP > 1 was used as the benchmark for screening key differentiating substances (Figure 5F).

**Figure 6:**
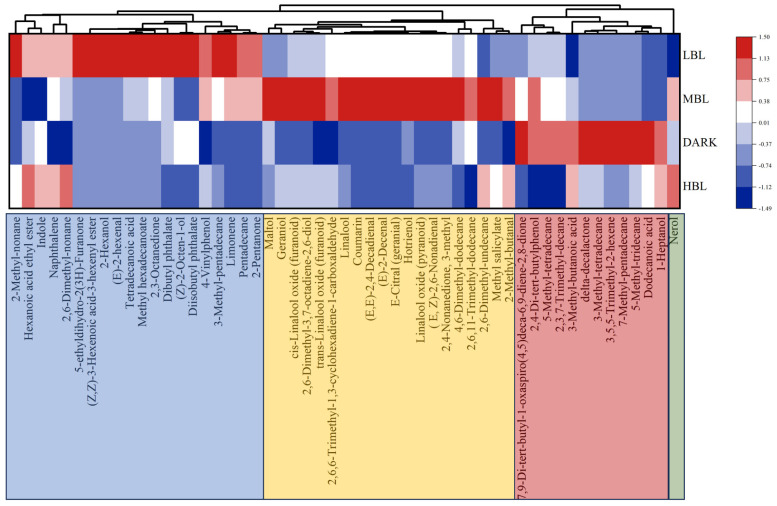
Analysis of 53 differential volatile compounds with VIP > 1 in green tea samples with different spreading light treatments.

A total of 53 volatile compounds ultimately fulfilled the VIP > 1 and *p* ≤ 0.05 conditions, indicating notable differences. The above 53 volatile compounds (Table S2) were divided into 4 categories according to the source: fatty acid-derived volatiles (FADVs, 32), amino acid-derived volatiles (AADVs, 5), volatile terpenoids (VTs, 7), carotenoid-derived volatiles (CDVs, 1), and others (8). Heatmaps were created to visually display how the content of these key volatile components changed during spreading under different light conditions, with red indicating upregulation and blue indicating downregulation [20]. In Figure 6, the first category, marked in blue, consisted of volatile compounds more connected to LBL treatment. The second category, marked in yellow (Figure 6), comprised volatile compounds were more linked to MBL treatment, such as geraniol and linalool. Volatile compounds more related to DARK treatment, such as 3-methyl-butanoic acid and dodecanoic acid, were included in the third category, shown in red in Figure 6. Nerol, which was more associated with HBL treatment, was part of the last category, marked in green in Figure 6.

### 3.4. Validation of Important Differential Volatile Compounds

**Figure 7:**
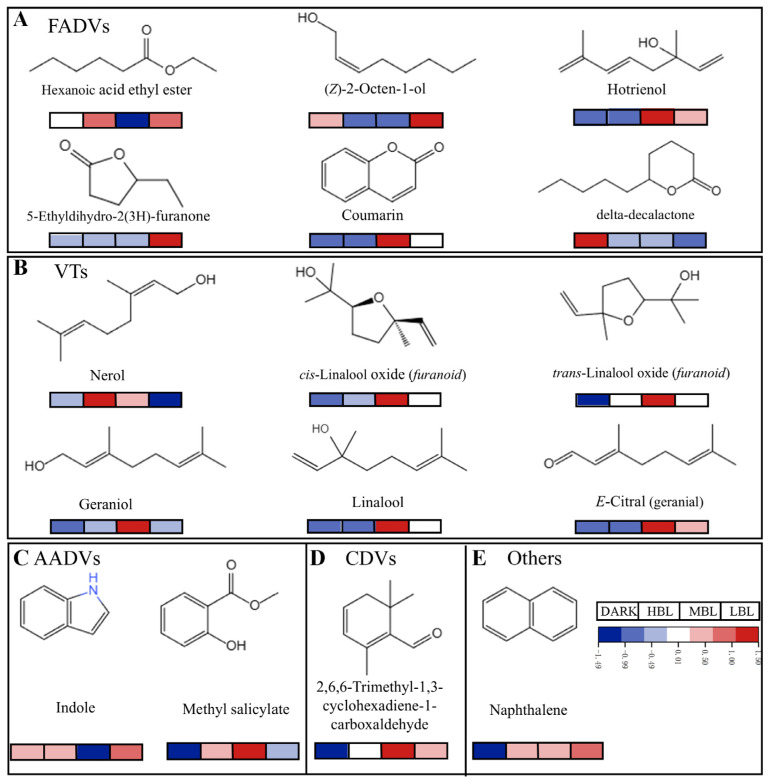
Effect of different light conditions on the contents of 16 important differential volatile compounds contributing to the aroma of green tea. (**A**) FADVs. (**B**) VTs. (**C**) AADVs. (**D**) CDVs. (**E**) Other sources of volatiles. Red indicates upregulation, and blue indicates downregulation.

A prior study indicated that the impact of aroma compounds in the system is influenced by the ratio of their concentration to the threshold [9]. Thus, we used the 53 compounds identified through OPLS-DA analysis in rOAV analysis to differentiate green tea samples exposed to various light conditions. Table 2 shows that 16 compounds were identified using GC-MS, each with rOAVs > 1. The rOAV of linalool exceeded 800, indicating a significant impact on the aroma, while hexanoic acid ethyl ester, geraniol, (*Z*)-2-octen-1-ol, naphthalene, and *E*-citral (geranial) (rOAV > 10), were identified as a key aroma component. The 16 volatile compounds that contribute significantly to the aroma of green tea under different light conditions during spreading were shown in Figure 7. MBL samples had the highest concentrations of coumarin and hotrienol from fatty acid-derived volatiles. The content of 5-ethyldihydro-2(3H)-furanone and (*Z*)-2-octen-1-ol were significantly increased under LBL treatment. Hexanoic acid ethyl ester significantly accumulated under HBL and LBL treatments, while delta-decalactone significantly accumulated under DARK treatment (Figure 7A). After spreading, the concentrations of geraniol, cis-linalool oxide (*furanoid*), *trans*-linalool oxide (*furanoid*), linalool, and *E*-citral (geranial) from volatile terpenoids were significantly higher in MBL samples, whereas levels of nerol were higher in HBL samples (Figure 7B). Indole and methyl salicylate were two amino acid-derived volatiles; indole significantly accumulated under LBL treatment, while methyl salicylate accumulated under MBL treatment (Figure 7C). In addition, the content of 2,6,6-trimethyl-1,3-cyclohexadiene-1-carboxaldehyde from carotenoid-derived volatiles was significantly higher in MBL than other treatments (Figure 7D). Naphthalene from other sources was accumulated only under increased blue light treatment after spreading (Figure 7E).

### 3.5. Relationship Network Analysis of Key Volatile Compounds

Pearson correlation analysis was performed to calculate correlation coefficients between variables. A network diagram was constructed to visualize intra-group correlations with absolute values exceeding the threshold of 0.8 and statistical significance (*p* < 0.05), revealing the internal structure of metabolite interactions. Nodes represent significantly differential metabolites, colored according to their regulation patterns: red for upregulated, blue for downregulated, and gray for non-differential metabolites. Edge colors denote interaction types, with red and blue lines representing positive and negative correlations, respectively. The edge thickness corresponds to the absolute magnitude of correlation coefficients, where thicker lines indicate stronger associations. This visualization strategy enables systematic identification of critical relationships between variables through characterization of both regulatory patterns and interaction strengths among significantly correlated metabolic features [22].

**Figure 8:**
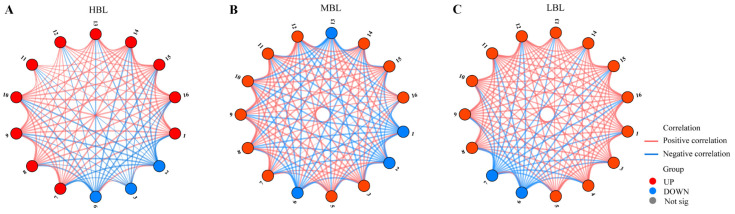
Interaction analysis of key volatile compounds based on correlation coefficients. (**A**) Analysis of interactions between key volatile compounds under HBL treatment during spreading. (**B**) Analysis of interactions between key volatile compounds under MBL treatment during spreading. (**C**) Analysis of interactions between key volatile compounds under LBL treatment during spreading. The numbers in the figure correspond to the serial numbers of the substances in Table 2.

The analysis of the relationship network diagram for key compounds in green tea under HBL treatment revealed that the main volatile compounds could be categorized into two groups (Figure 8A). (*Z*)-2-Octen-1-ol, hotrienol and delta-decalactone in the first group of compounds showed a negative correlation with other compounds. Nerol, *cis*-linalool oxide (*furanoid*), *trans*-linalool oxide (*furanoid*), geraniol, linalool, *E*-citral (geranial), indole, methyl salicylate, 2,6,6-trimethyl-1,3-cyclohexadiene-1-carboxaldehyde, and naphthalene in the second group of compounds showed a positive correlation with other compounds. Hexanoic acid ethyl ester, (*Z*)-2-octen-1-ol, and delta-decalactone were negatively correlated with other substances in MBL treatment. Hotrienol, coumarin, nerol, *cis*-linalool oxide (*furanoid*), *trans*-linalool oxide (*furanoid*), geraniol, linalool, *E*-citral (geranial), methyl salicylate, 2,6,6-trimethyl-1,3-cyclohexadiene-1-carboxaldehyde, and naphthalene were positively correlated with other substances in MBL treatment. Nerol and delta-decalactone were negatively correlated with other substances in LBL treatment. Hexanoic acid ethyl ester, hotrienol, 5-ethyldihydro-2(3H)-furanone, coumarin, *cis*-linalool oxide (*furanoid*), *trans*-linalool oxide (*furanoid*), geraniol, linalool, *E*-citral (geranial), indole, methyl salicylate, 2,6,6-trimethyl-1,3-cyclohexadiene-1-carboxaldehyde, and naphthalene were positively correlated with other substances in LBL treatment.

## 4. Discussion

Tea is the second most widely consumed drink, surpassed only by water, in many societies [23]. Around the globe, its special flavor, fragrance, and health advantages are highly prized [24]. Typically, green tea quality is assessed by examining its color, aroma, taste, shape, and the appearance of its steeped leaves. The type of aroma is a crucial aspect of green tea quality, significantly influencing its market price and consumer preference [25]. Green tea’s scent [19] can be categorized into different types, such as floral, sweet, chestnut-like, and fresh aromas. Spreading, fixation, rolling, and drying all impact flavor; spreading receives more focus. As spreading occurs, volatile compounds, amino acids, and glycosides experience notable changes, which may lead to an increase in amino acids, suppression of catechin and flavonoid production, and a decrease in the phenol-to-amine ratio [26].

Part of the aroma components of tea are inherent in fresh leaves, and the other part is mainly formed during processing [27]. The initial step in processing green tea, spreading, has a direct impact on the tea quality. After spreading, the leaves start to wilt, their texture shifts from firm to soft, their color transitions from bright green to dark green, and ultimately, the leaves lose their greenness entirely. The aroma is also released, and the moisture level of the spread leaves decreases to 70 ± 2%, which is a moderate level, setting the stage for the next step in the fixation process [28]. In traditional processing, solar withering is widely used in the manufacturing process of tea to improve the overall quality. Indoor trough withering compensates for the environmental drawbacks in natural withering and enhances both efficiency and quality [5].

At present, the positive effects of different light conditions on tea quality have been confirmed, so the installation of LED lamps in traditional indoor withering chambers has been applied. The content and composition of volatile compounds are easily affected by the light conditions during different withering conditions. Red light and UV-B withering could enhance the activity and up-regulate the expression of *β*-primeverosidase and β-GLU during withering, leading to the release of free GBVs (glycosides), such as linalool and its oxides [13,29]. During the green tea spreading process, the red light was beneficial to accumulate components that produce a chestnut-like aroma [30]. Linalool synthase (*CsLIS2*, *CsLIS3*, *CsLIS4*) and farnesene synthase (*CsFS5*, *CsFS10*) were significantly up-regulated in spreading leaves after LTY (low temperature + yellow light) treatment and significantly improved the aroma quality of green tea products [31].

The pleasant scents of green tea are frequently characterized as tender and fresh, with notes like chestnuts and orchids. The unpleasant scents of green tea are frequently characterized as grassy, dull, and over-fired. An experienced sensory panel can subjectively differentiate these various aroma types, or they can be objectively distinguished using analytical instruments and chemometric techniques [32]. In the MBL treatment, the floral aroma was significantly more intense than in the DARK treatment, which might be attributed to higher linalool concentrations. Furthermore, the greater amounts of nerol might contribute to the stronger fruity aroma in HBL treatment. This discovery aligned with related studies, indicating that blue light treatment was the most effective in enhancing the floral aroma [12].

The qualitative and quantitative analysis showed notable variations in the aroma components of the four green tea samples when exposed to different light conditions during spreading. In this study, MBL treatment increased both the total content and the variety of aroma compounds, suggesting that it enhanced the aroma intensity of green tea. This could primarily be due to variations in the quantity of aroma precursors and enzyme activity influenced by light conditions during spreading [9]. In addition, light treatment also impacted the volatile compounds’ composition and content during spreading. MBL treatment led to a significant rise in alcohols and aldehydes, while HBL treatment caused a significant increase in ketones. In a similar manner, earlier studies indicated that blue light treatment, as opposed to DARK treatment, notably raised the levels of aldehydes, ketones and alcohols in black tea [33].

In plants, typical aromatic precursors are lipids, amino acids, geranyl/farnesyl pyrophosphate, and carotenoids, which can be converted into volatile compounds via the metabolic processes of lipid oxidation, amino acid breakdown, terpenoid synthesis, and carotenoid breakdown, respectively [34]. Therefore, the volatile compounds in tea can be categorized as FADVs, AADVs, VTs, and CDVs [35]. During withering, fresh leaves in vitro continue to exhibit strong synthesis, transformation, and metabolism of FADVs, AADVs, VTs, and CDVs [8,36]. In this study, 16 important differential compounds were identified, including 6 FADVs, 6 VTs, 2 AADVs, 1 CDV, and 1 other. This indicated that FADVs and VTs had major contribution to the formation of green tea aroma under different blue light intensity treatments.

Hexanoic acid ethyl ester, 5-ethyldihydro-2(3H)-furanone, hotrienol, delta-decalactone, coumarin, and (*Z*)-2-octen-1-ol were recognized as primary FADV aroma compounds due to its high rOAVs. Hotrienol, (*Z*)-2-octen-1-ol, delta-decalactone, and hexanoic acid ethyl ester were volatile fatty acid derivatives with green or grass odor. Under LBL and DARK treatment, the above three compounds accumulated in green tea, which might lead to the green and grass-like odor in green tea. Coumarin is a natural substance with a sweet, herb-like, and cherry blossom scent. It has been identified as a key compound contributing to the sweet aroma of Japanese and Chinese green tea [37]. Green tea treated with MBL had a notably higher coumarin content compared to other treatment groups, enhancing its sweet aroma of green tea. Furaneol, which imparts ’sweet, fruity, and caramel’ notes, is extensively found in berry fruits, like strawberry [6]. In Laoshan green tea, furaneol was identified as a key aroma compound [9]. 5-Ethyldihydro-2(3H)-furanone was only accumulated under LBL treatment, which may be the source of the sweet aroma of green tea under LBL treatment.

Geraniol, *cis*-linalool oxide (*furanoid*), *trans*-linalool oxide (*furanoid*), nerol, linalool, and *E*-citral (geranial) were identified as the key VTs of aroma. The “fruit sweet fragrance” of green tea was mainly composed of linalool, linalool oxide, geraniol, and nerol [31]. Linalool has been extensively researched and found to be the compound that imparts a chestnut-like aroma [19]. Geraniol is a colorless oily liquid with a rose-like scent, and it is often detected in green tea. Geraniol has been reported to exhibit positive correlations with the Huangshan Maofeng grade [38]. Linalool, linalool oxide, and geraniol were found to accumulate under MBL treatment, possibly leading to the fruity and sweet aroma in green tea. In sunlight withering, the increased activity of geranyl pyrophosphate synthase and alcohol dehydrogenase was associated with an enhanced floral aroma due to the enrichment of terpene-catalyzed products and benzeneacetaldehyde concentration [39]. MBL treatment with green tea may reduce isopentenyl pyrophosphate and dimethylallyl pyrophosphate concentrations, resulting in elevated levels of linalool and geraniol. Geraniol can be further converted to nerol, neral, and orcitral during green tea spreading [40]. Similarly, nerol accumulated under HBL conditions during spreading, suggesting that most geraniol was converted into nerol under HBL conditions.

According to previous research, indole (floral, animal-like) was believed to give a unique aroma to both Japanese green tea (Sen-cha) [41] and oolong tea [42]. In addition, indole [25] contributed to the chestnut-like aroma in green tea; it accumulates during LBL treatment, possibly serving as the floral and chestnut-like source. Methyl salicylate has a minty scent and has been found in green, oolong, and black tea [40]. Methyl salicylate accumulated under MBL treatment during spreading, which may be the sweet flavor source of green tea under MBL treatment. Characterized by its unique and sharp naphthyl odors, naphthalene is a polycyclic aromatic hydrocarbon formed from long-chain hydrocarbons when exposed to high temperatures [18]. In addition, CDV 2,6,6-trimethyl-1,3-cyclohexadiene-1-carboxaldehyde (woody, spicy, medicinal, powdery, herbal) was detected. The effect of 2,6,6-trimethyl-1,3-cyclohexadiene-1-carboxaldehyde on MBL treatment increases the refreshing aroma of green tea.

## 5. Conclusions

The intensity of blue light significantly influenced the sensory quality and aroma development in green tea. This research systematically examined the volatile components of green tea subjected to various light treatments during the spreading stage using HS-SPME-GC-MS. MBL was identified as the optimal light condition for creating high-quality green tea with a robust and enduring fruity and flower aroma. OPLS-DA and rOAV analysis identified the 16 main volatile compounds. Blue light intensity significantly impacted different sources of volatile compounds; MBL treatment being the most effective in forming and transforming volatile terpenes, thereby enhancing the fruity and floral aroma in green tea. Enhancing the nerol content through HBL treatment improved the flower aroma in green tea. To provide technical guidance to produce high-quality green tea, the influence of different light conditions on the formation of aroma during tea spreading was systematically studied. The research results provide a theoretical basis and technical support for the targeted processing of high-quality green tea.

## Figures and Tables

**Table 1:** The details of volatile compounds identified by GC-MS.

No.	Name	Rt	RI	Content (μg/kg, DW)			
				HBL	MBL	LBL	DARK
**Alcohols**							
1	1-Penten-3-ol	2.33	684	256.62 ± 0.25 a	186.79 ± 0.39 b	141.71 ± 0.99 c	134.71 ± 2.41 d
2	1-Pentanol	3.42	765	70.62 ± 0.23 a	38.86 ± 0.55 b	20.71 ± 0.11 d	25.61 ± 0.07 c
3	2-Hexanol	3.99	803	N.d	N.d	17.62 ± 1.05 a	N.d
4	(*Z*)-3-Hexen-1-ol	5.05	857	108.04 ± 1.74 a	77.22 ± 0.49 b	51.54 ± 0.92 c	46.21 ± 1.16 d
5	1-Hexanol	5.25	868	27.6 ± 0.42 a	15.5 ± 0.19 c	16.49 ± 0.43 b	8.51 ± 0.22 d
6	1-Heptanol	7.17	970	6.88 ± 0.04 b	N.d	N.d	9.42 ± 0.31 a
7	1-Octen-3-ol	7.38	980	354.33 ± 2.96 b	162.18 ± 1.34 d	237.21 ± 1.35 c	393.34 ± 2.37 a
8	2-Ethyl-1-hexanol	8.28	1030	N.d	N.d	10.41 ± 0.33 b	13.32 ± 0.51 a
9	Benzyl alcohol	8.40	1036	309.14 ± 0.43 b	327.76 ± 0.32 a	190.82 ± 1.19 c	113.32 ± 2.26 d
10	(*Z*)-2-Octen-1-ol	9.06	1067	N.d	N.d	36.12 ± 22.15 a	17.82 ± 0.64 ab
11	(*E*)-2-Octen-1-ol	9.10	1068	86.11 ± 1.07 a	N.d	N.d	N.d
12	*cis*-Linalool oxide (*furanoid*)	9.20	1074	140.99 ± 1.58 c	225.52 ± 0.37 a	156.12 ± 2.88 b	104.9 ± 1.78 d
13	*trans*-Linalool oxide (*furanoid*)	9.53	1086	462.35 ± 2.12 c	668.91 ± 0.6 a	477.59 ± 1.34 b	313.93 ± 4.15 d
14	Linalool	9.76	1099	188.1 ± 1.87 c	415.93 ± 0.85 a	285.27 ± 2.26 b	175.16 ± 4.36 d
15	Hotrienol	9.87	1107	57.05 ± 1.16 d	114.65 ± 0.96 a	87.26 ± 1.91 b	62.2 ± 1.34 c
16	Phenylethyl alcohol	10.10	1116	912.04 ± 1.7 b	1003.42 ± 0.97 a	648.07 ± 8.75 c	465.13 ± 4.37 d
17	2,6-Dimethyl-3,7-octadiene-2,6-diol	11.82	1190	73.01 ± 0.17 b	114.74 ± 0.57 a	75.75 ± 2.03 b	46.18 ± 0.95 c
18	4-Vinylphenol	12.79	1219	36.54 ± 0.76 c	66.58 ± 2.14 b	82.98 ± 4.71 a	N.d
19	Nerol	12.97	1228	58.21 ± 0.89 a	42.19 ± 1.49 b	N.d	24.77 ± 0.03 c
20	Geraniol	13.63	1255	1152.5 ± 0.06 b	1613.6 ± 3.57 a	1107.96 ± 7.29 c	1016.93 ± 1.58 d
21	Phytol	36.06	2114	N.d	56.95 ± 0.99 c	155.1 ± 0.57 a	146.9 ± 4.37 b
**Aldehydes**							
22	3-Methyl-butanal	2.12	652	53.01 ± 2.41 a	38.4 ± 0.24 b	27.23 ± 0.05 c	30.31 ± 0.84 c
23	2-Methyl-butanal	2.19	662	62.69 ± 0.24 b	66.55 ± 0.61 a	56.6 ± 0.9 c	52.04 ± 1.38 d
24	Hexanal	3.91	800	138.63 ± 0.27 a	N.d	N.d	19.48 ± 0.32 b
25	(*E*)-2-hexenal	4.67	854	N.d	N.d	7.89 ± 1.84 a	N.d
26	Benzaldehyde	7.09	962	76.1 ± 0.11 a	35.66 ± 0.49 b	16.75 ± 0.19 c	9.53 ± 0.19 d
27	(*E*,*E*)-2,4-Heptadienal	7.91	1011	N.d	8.44 ± 2.2 a	8.68 ± 2.13 a	N.d
28	Benzeneacetaldehyde	8.62	1045	77.95 ± 2.02 a	30.1 ± 0.98 b	19.53 ± 0.43 c	13.34 ± 1.09 d
29	(*E*)-2-octenal	8.83	1060	115.52 ± 2.78 a	N.d	N.d	N.d
30	(*E*,*Z*)-2,6-Nonadienal	11.00	1155	3.44 ± 0.18 c	34.77 ± 0.92 a	17.72 ± 0.34 b	N.d
31	2,6,6-Trimethyl-1,3-cyclohexadiene-1-carboxaldehyde	12.18	1201	18.59 ± 0.25 c	23.33 ± 0.33 a	20.52 ± 0.35 b	14.61 ± 0.07 d
32	Decanal	12.24	1205	69.86 ± 0.5 a	44.91 ± 1.78 b	35.31 ± 1.55 c	N.d
33	β-Cyclocitral	12.81	1220	22.98 ± 0.7 b	27.13 ± 1.37 a	17.54 ± 0.42 c	9.58 ± 0.16 d
34	Neral	13.29	1240	64.65 ± 14.74 a	62.12 ± 10.81 a	42.64 ± 1.09 a	N.d
35	(*E*)-2-Decenal	13.83	1263	2.83 ± 0.3 c	246.31 ± 3.32 a	127.05 ± 1.27 b	N.d
36	*E*-Citral (geranial)	14.09	1271	2.97 ± 1.07 c	484.22 ± 0.79 a	262.97 ± 7.74 b	N.d
37	(*E*,*E*)-2,4-Decadienal	15.02	1317	N.d	90.79 ± 23.27 a	47.01 ± 2.38 b	N.d
38	Vanillin	17.82	1405	70.41 ± 2.51 a	11.55 ± 1.38 b	N.d	N.d
**Acids**							
39	3-Methyl-butanoic acid	4.70	863	29.45 ± 0.54 b	24.55 ± 0.15 c	18.64 ± 0.18 d	31.14 ± 0.24 a
40	2-Methyl-butanoic acid	4.89	861	N.d	8.54 ± 0.33 c	14.78 ± 0.2 b	20.29 ± 0.09 a
41	Pentanoic acid	5.49	904	36.06 ± 6.57 a	N.d	N.d	N.d
42	n-Decanoic acid	16.84	1373	54.5 ± 9.54 a	43.49 ± 5.97 a	N.d	N.d
43	Dodecanoic acid	22.46	1568	112.08 ± 2.99 b	N.d	N.d	246.7 ± 3.32 a
44	Tetradecanoic acid	27.64	1769	N.d	12.31 ± 1.33 b	77.86 ± 4.08 a	N.d
**Esters**							
45	Ethyl Acetate	1.87	612	18.88 ± 0.57 c	78.78 ± 0.29 a	68.33 ± 0.6 b	67.52 ± 0.93 b
46	Hexanoic acid ethyl ester	7.76	1000	12.62 ± 0.02 a	N.d	12.24 ± 0.44 a	6.9 ± 0.1 b
47	(*E*)-3-Hexenoic acid ethyl ester	7.86	1006	3.27 ± 0.59 a	N.d	N.d	N.d
48	Methyl salicylate	12.04	1192	142.13 ± 2.45 b	174.14 ± 1.5 a	118.26 ± 0.83 c	95.51 ± 2.05 d
49	(*Z*)-Hexanoic acid-3-hexenyl ester	17.21	1380	N.d	287.95 ± 0.89 b	434.35 ± 1.11 a	181.14 ± 7.63 c
50	(*Z*,*Z*)-3-Hexenoic acid-3-hexenyl ester	17.34	1389	N.d	N.d	80.74 ± 0.43 a	N.d
51	Diethyl phthalate	23.16	1594	173.09 ± 2.28 a	26.16 ± 0.32 b	23.53 ± 0.35 b	N.d
52	Methyl jasmonate	23.63	1648	32.53 ± 0.49 a	N.d	N.d	N.d
53	2-Ethylhexyl-benzoic acid ester	26.37	1735	N.d	127.54 ± 2.1 a	86.27 ± 0.77 b	24.84 ± 0.55 c
54	Diisobutyl phthalate	30.25	1870	64.64 ± 1.62 c	66.14 ± 0.28 c	120.69 ± 0.6 a	95.54 ± 2.69 b
55	Methyl hexadecanoate	31.74	1926	N.d	12.16 ± 1 b	50.61 ± 2.12 a	N.d
56	Dibutyl phthalate	32.62	1965	53.48 ± 0.98 c	90.26 ± 0.07 b	213.87 ± 1.48 a	95.65 ± 3.36 b
57	Ethyl hexadecanoate	33.38	1993	31.37 ± 0.29 c	71.89 ± 1.56 b	74 ± 1.72 b	92.84 ± 1.69 a
58	(*E*)-2-Hexenyl-hexanoic acid ester	17.43	1391	N.d	46.23 ± 1.31 c	95.82 ± 4.6 a	59.72 ± 0.63 b
59	Delta-decalactone	20.48	1495	488.87 ± 7.27 b	449.06 ± 0.77 b	253.4 ± 5.09 c	1555.69 ± 36.95 a
**Ketons**							
60	2-Pentanone	1.95	655	N.d	49.92 ± 1.08 b	61.15 ± 1.36 a	N.d
61	2-Heptanone	5.72	891	23.37 ± 4.65 a	N.d	N.d	N.d
62	2,3-Octanedione	7.42	985	N.d	13.04 ± 2.33 b	34.42 ± 3.29 a	N.d
63	6-Methyl-5-hepten-2-one	7.54	986	N.d	25.94 ± 0.28 c	35.68 ± 0.17 a	30.02 ± 0.59 b
64	(*E*)-3-Octen-2-one	8.42	1033	77.09 ± 0.58 a	N.d	N.d	N.d
65	2,2,6-Trimethyl-cyclohexanone	8.42	1036	N.d	12.7 ± 0.13 b	16.93 ± 0.37 a	12.45 ± 0.2 b
66	(*R*,*S*)-5-Ethyl-6-methyl-3 E-hepten-2-one	10.78	1144	58.67 ± 0.39 a	18.48 ± 0.44 b	N.d	N.d
67	α-Ionone	18.47	1426	76.29 ± 2.63 a	N.d	N.d	N.d
68	Geranylacetone	19.14	1456	295.4 ± 6.23 a	N.d	N.d	N.d
69	*trans*-*β*-Ionone	20.24	1486	762.8 ± 4.48 a	656.47 ± 1.52 b	336.95 ± 3.21 c	225.39 ± 4.8 d
70	*cis*-Jasmone	17.76	1394	N.d	105.61 ± 1.04 b	144.78 ± 1.72 a	83.82 ± 1.29 c
71	2,4-Nonanedione, 3-methyl	13.45	1252	11.23 ± 0.42 c	64.17 ± 1.04 a	33.91 ± 0.37 b	N.d
**Hydrocarbons**							
72	2-Methyl-nonane	7.11	964	4.62 ± 0.18 b	N.d	8.28 ± 0.09 a	N.d
73	3,5,5-Trimethyl-2-hexene	7.30	985	N.d	N.d	N.d	137.75 ± 3.24 a
74	2,6-Dimethyl-nonane	8.11	1018	51.67 ± 1.28 a	42.02 ± 1.06 c	47.08 ± 2.13 b	34.2 ± 0.67 d
75	Limonene	8.30	1030	N.d	8.43 ± 0.87 b	13.6 ± 1.28 a	N.d
76	β-Ocimene	8.67	1037	N.d	22.82 ± 0.9 a	15.88 ± 0.89 b	N.d
77	2,9-Dimethyl-decane	10.32	1126	9.01 ± 0.25 a	N.d	N.d	N.d
78	Naphthalene	11.94	1181	16.14 ± 0.61 a	15.54 ± 2.3 a	18.38 ± 0.58 a	N.d
79	Dodecane	12.10	1200	72.23 ± 0.87 b	89.51 ± 0.05 a	65.68 ± 0.33 c	36.18 ± 3.07 d
80	2,4-Dimethyl-undecane	12.38	1208	28.71 ± 0.49 a	26.16 ± 0.73 b	15.81 ± 0.26 c	15.51 ± 0.47 c
81	2,6-Dimethyl-undecane	12.56	1210	141.53 ± 1.95 b	166.62 ± 2.12 a	91.79 ± 0.62 c	91.17 ± 1.11 c
82	4-Methyl-dodecane	13.66	1259	55.24 ± 15.05 a	N.d	N.d	N.d
83	2,3-Dimethyl-undecane	13.77	1251	261.58 ± 7.56 a	N.d	N.d	N.d
84	2,6,11-Trimethyl-dodecane	14.49	1275	N.d	103.41 ± 20.41 a	60.42 ± 0.68 b	61.31 ± 1.02 b
85	Tridecane	14.80	1300	8.25 ± 0.29 c	86.36 ± 1.42 a	58.8 ± 0.6 b	55.54 ± 3.06 b
86	4,6-Dimethyl-dodecane	15.34	1325	62.48 ± 14.8 b	199.48 ± 42.43 a	117.46 ± 0.93 b	117.59 ± 0.97 b
87	6-Methyl-tridecane	16.32	1344	N.d	28.46 ± 5.12 b	28.17 ± 9.44 b	93.88 ± 5.15 a
88	5-Methyl-tridecane	16.40	1348	24.41 ± 1.08 b	N.d	N.d	73.49 ± 1.12 a
89	4-Methyl-tridecane	16.45	1359	N.d	28.08 ± 1.17 c	32.69 ± 0.87 b	78.63 ± 1.43 a
90	3-Methyl-tridecane	16.79	1371	33.54 ± 1.97 b	41.28 ± 7.66 b	45.18 ± 20.79 b	154.67 ± 4.73 a
91	5-Methyl-tetradecane	19.24	1453	N.d	224.51 ± 9.47 b	144.59 ± 2.53 c	322.47 ± 3.48 a
92	4-Methyl-tetradecane	19.39	1459	511.39 ± 6.69 a	160.73 ± 0.92 c	129.95 ± 5.4 d	253.77 ± 0.8 b
93	2,3,7-Trimethyl-decane	19.51	1466	88.81 ± 0.75 d	665.63 ± 6.45 b	504.4 ± 5.8 c	967.42 ± 8.51 a
94	3-Methyl-tetradecane	19.64	1470	83.38 ± 2.24 b	N.d	N.d	440.51 ± 30.12 a
95	Pentadecane	20.56	1500	N.d	605.15 ± 2.9 b	794.43 ± 14.97 a	N.d
96	7-Methyl-pentadecane	21.90	1541	N.d	N.d	N.d	192.88 ± 5 a
97	3-Methyl-pentadecane	22.54	1570	16.44 ± 1.08 c	163.85 ± 12.13 b	308.29 ± 22.13 a	N.d
98	Hexadecane	23.26	1600	N.d	191.45 ± 1.56 c	502.66 ± 2.23 b	628.56 ± 6.06 a
99	4-Methyl-hexadecane	23.84	1659	18.56 ± 0.32 a	N.d	N.d	N.d
100	Neophytadiene	29.65	1837	N.d	15.22 ± 0.27 c	35.48 ± 0.43 a	25.33 ± 0.53 b
**Heterocyclic**							
101	2-Ethyl-furan	2.52	703	28.02 ± 0.69 a	14.79 ± 0.24 b	7.14 ± 0.21 c	8.45 ± 0.54 c
102	1-Ethyl-1H-pyrrole	4.28	821	N.d	12.58 ± 0.21 a	10.27 ± 0.14 b	12.72 ± 0.97 a
103	Butyrolactone	6.11	915	23.88 ± 0.07 a	12.36 ± 0.5 b	N.d	N.d
104	2-Pentylfuran	7.63	993	163.98 ± 0.62 a	93.09 ± 1.44 b	53.49 ± 1.21 c	27.51 ± 1.12 d
105	2,3,5-Trimethylpyrazine	7.86	1008	26.75 ± 7.28 a	N.d	N.d	N.d
106	5-ethyldihydro-2(3H)-Furanone	8.73	1057	N.d	N.d	23.4 ± 0.34 a	N.d
107	2-Acetyl pyrrole	8.86	1064	29.41 ± 1.12 c	176.02 ± 0.93 a	177.28 ± 0.66 a	147.3 ± 4.19 b
108	Maltol	10.08	1110	N.d	56.07 ± 0.92 a	N.d	5.51 ± 0.05 b
109	Linalool oxide (*pyranoid*)	11.45	1173	325.7 ± 0.4 c	558.02 ± 0.43 a	424.04 ± 2.91 b	289.31 ± 2.25 d
110	Indole	14.78	1295	67.68 ± 1.43 a	N.d	70.67 ± 0.28 a	58.04 ± 1.76 b
111	Coumarin	18.87	1441	34.7 ± 1.9 c	53.87 ± 0.7 a	43.14 ± 0.81 b	34.9 ± 0.28 c
112	Dihydroactinolide	21.46	1538	605.45 ± 3.91 a	462.74 ± 2.27 b	264.46 ± 4.66 d	373.34 ± 2.61 c
113	7,9-Di-tert-butyl-1-oxaspiro(4,5)deca-6,9-diene-2,8-dione	31.60	1923	20.25 ± 0.67 c	25.68 ± 0.15 b	21.35 ± 0.01 c	33.01 ± 1.35 a
**Others**							
114	1,1-Diethoxy-ethane	2.81	726	222.43 ± 0.52 a	10.62 ± 0.28 b	N.d	9.04 ± 0.99 b
115	p-Vinylguaiacol	14.86	1317	114.84 ± 1.92 a	N.d	N.d	N.d
116	2,4-Di-tert-butylphenol	20.94	1519	107.69 ± 0.83 c	256.55 ± 2.91 a	176.8 ± 0.88 b	251.01 ± 5.36 a

DW, dry weight. RT, Retention time. RI, Retention index calculated from a series of n-alkanes (C7-C40). N.d indicates lower than the instrument detection limit. Different letters in the same column indicate a significant difference between light conditions (*p* < 0.05).

**Table 2:** Aroma compounds identified in green teas under different light conditions.

No.	Compound Name	Odor Characteristic	OTs (ug/kg)	rOAV			
				HBL	MBL	LBL	DARK
1	Hexanoic acid ethyl ester	citrus, green	0.7	18.03 ± 0.03 a	N.d	17.49 ± 0.63 a	9.86 ± 0.14 b
2	(*Z*)-2-Octen-1-ol	Sweet, green, citrus, fatty, herbal, cucumber- like	3	N.d	N.d	12.04 ± 7.38 a	5.94 ± 0.21 ab
3	Hotrienol	Fresh, floral, fruity	110	0.52 ± 0.01 d	1.04 ± 0.01 a	0.79 ± 0.02 b	0.57 ± 0.01 c
4	5-Ethyldihydro-2(3 H)-furanone	Caramel, nutty, roasted, sweet, creamy	9.7	N.d	N.d	2.41 ± 0.03 a	N.d
5	Coumarin	Floral	11	3.15 ± 0.17 c	4.9 ± 0.06 a	3.92 ± 0.07 b	3.17 ± 0.03 c
6	delta-Decalactone	Green, herbal	66	7.41 ± 0.11 b	6.8 ± 0.01 b	3.84 ± 0.08 c	23.57 ± 0.56 a
7	Nerol	Fresh, citrus, floral, green, sweet, lemon-like	49	1.19 ± 0.02 a	0.86 ± 0.03 b	N.d	0.51 ± 0.00 c
8	*cis*-Linalool oxide (*furanoid*)	Sweet, floral, creamy	190	0.74 ± 0.01 c	1.19 ± 0.00 a	0.82 ± 0.02 b	0.55 ± 0.01 d
9	*trans*-Linalool oxide (*furanoid*)	Sweet, floral, creamy	190	2.43 ± 0.01 c	3.52 ± 0.00 a	2.51 ± 0.01 b	1.65 ± 0.02 d
10	Geraniol	Rose-like, sweet, honey-like	7.5	153.67 ± 0.01 b	215.15 ± 0.48 a	147.73 ± 0.97 c	135.59 ± 0.21 d
11	Linalool	Floral, sweet, grape-like, woody	0.22	854.99 ± 8.48 c	1890.6 ± 3.86 a	1296.68 ± 10.27 b	796.2 ± 19.8 d
12	*E*-Citral (geranial)	Citrus, lemon-like	1	2.97 ± 1.07 c	484.22 ± 0.79 a	262.97 ± 7.74 b	N.d
13	Indole	Floral, animal-like	40	1.69 ± 0.04 a	N.d	1.77 ± 0.01 a	1.45 ± 0.04 b
14	Methyl salicylate	Minty, wintergreen-like	40	3.55 ± 0.06 b	4.35 ± 0.04 a	2.96 ± 0.02 c	2.39 ± 0.05 d
15	2,6,6-Trimethyl-1,3-cyclohexadiene-1-carboxaldehyde	Woody, spicy, medicinal, powdery, herbal	3	6.20 ± 0.08 c	7.78 ± 0.11 a	6.84 ± 0.12 b	4.87 ± 0.02 d
16	Naphthalene	Pungent, tar	0.44	36.68 ± 1.39 a	35.32 ± 5.22 a	41.77 ± 1.32 a	N.d

OTs: odor threshold in water based on the literature [21]. Relative odor activity value (rOAV), calculated as the ratio of odorant relative concentration in the green tea to odor thresholds in water. N.d indicates lower than the instrument detection limit. Different letters in the same column indicate a significant difference between light conditions (*p* < 0.05).

## Data Availability

The original contributions presented in the study are included in the article; further inquiries can be directed to the corresponding author.

## Supplementary Materials

The following supporting information can be downloaded at: https://www.mdpi.com/article/10.3390/foods14081308/s1, Table S1: Volatile compounds were detected by HS-SPME-GC-MS in green tea under different light conditions; Table S2: Volatile components screened according to VIP > 1 and *p* < 0.05 in green tea under different light conditions.

## Abbreviations

The following abbreviations are used in this manuscript:

AADVs	Amino acid-derived volatiles
CDVs	Carotenoid-derived volatiles.
DARK	Dark treatment
FADVs	Fatty acid-derived volatiles
GC-MS	Gas chromatography-mass spectrometry
HBL	High-intensity blue light treatment
HCA	Hierarchical clustering analysis
HS-SPME	Headspace solid-phase microextraction
LBL	Low-intensity blue light treatment
LED	Light emitting diode
MBL	Middle-intensity blue light treatment
NIST	National Institute of Standards and Technology
OPLS-DA	Orthogonal partial least squares discriminant analysis
PCA	Principal component analysis
rOAV	Relative odor activity value
VIP	Variables important in the projection
VTs	Volatile terpenoids

## References

[1] Sheibani E., Duncan S.E., Kuhn D.D., Dietrich A.M., O’Keefe S.F. (2016). SDE and SPME Analysis of Flavor Compounds in Jin Xuan Oolong Tea. J. Food Sci..

[2] Yin P., Kong Y., Liu P., Wang J., Zhu Y., Wang G., Sun M., Chen Y., Guo G., Liu Z. (2022). A critical review of key odorants in green tea: Identification and biochemical formation pathway. Trends Food Sci. Technol..

[3] Yang Y.-Q., Yin H.-X., Yuan H.-B., Jiang Y.-W., Dong C.-W., Deng Y.-L. (2018). Characterization of the volatile components in green tea by IRAE-HS-SPME/GC-MS combined with multivariate analysis. PLoS ONE.

[4] Kaczyński P., Iwaniuk P., Jankowska M., Orywal K., Socha K., Perkowski M., Farhan J.A., Łozowicka B. (2024). Pesticide residues in common and herbal teas combined with risk assessment and transfer to the infusion. Chemosphere.

[5] Qi D., Shi Y., Lu M., Ma C., Dong C. (2024). Effect of withering/spreading on the physical and chemical properties of tea: A review. Comp. Rev. Food Sci. Food Safe.

[6] Ho C., Zheng X., Li S. (2015). Tea aroma formation. Food Sci. Hum. Wellness.

[7] Lasekan O., Lasekan A. (2012). Flavour chemistry of mate and some common herbal teas. Trends Food Sci. Technol..

[8] Chen Q., Shi J., Mu B., Chen Z., Dai W., Lin Z. (2020). Metabolomics combined with proteomics provides a novel interpretation of the changes in nonvolatile compounds during white tea processing. Food Chem..

[9] Zhu J., Niu Y., Xiao Z. (2021). Characterization of the key aroma compounds in Laoshan green teas by application of odour activity value (OAV), gas chromatography-mass spectrometry-olfactometry (GC-MS-O) and comprehensive two-dimensional gas chromatography mass spectrometry (GC × GC-qMS). Food Chem..

[10] Sui M., Wang L., Xue R., Xiang J., Wang Y., Yuan Y., Pu Q., Fang X., Liu B., Hu X. (2024). The aroma formation from fresh tea leaves of Longjing 43 to finished *Enshi Yulu* tea at an industrial scale. J. Sci. Food Agric..

[11] Ouzounis T., Rosenqvist E., Ottosen C.-O. (2015). Spectral Effects of Artificial Light on Plant Physiology and Secondary Metabolism: A Review. HortScience.

[12] Hua J., Zhu X., Ouyang W., Yu Y., Chen M., Wang J., Yuan H., Jiang Y. (2024). Non-target and target quantitative metabolomics with quantitative aroma evaluation reveal the influence mechanism of withering light quality on tea aroma and volatile metabolites evolution. Food Res. Int..

[13] Li Y., He C., Yu X., Zhou J., Ntezimana B., Yu Z., Chen Y., Ni D. (2022). Study on improving aroma quality of summer-autumn black tea by red-light irradiation during withering. LWT.

[14] Lin J., Liu F., Zhou X., Tu Z., Chen L., Wang Y., Yang Y., Wu X., Lv H., Zhu H. (2022). Effect of red light on the composition of metabolites in tea leaves during the withering process using untargeted metabolomics. J. Sci. Food Agric..

[15] Mu L., Li T., Tang J., Liu L., Wang R. (2021). Effects of LED Light Withering on the Quality of White Tea. IOP Conf. Ser. Earth Environ. Sci..

[16] Xie J., Wang Q., Hu J., Wang L., Yu X., Yuan H., Jiang Y., Yang Y. (2025). Uncovering the effects of spreading under different light irradiation on the volatile and non-volatile metabolites of green tea by intelligent sensory technologies integrated with targeted and non-targeted metabolomics analyses. Food Chem..

[17] Zhu Y.-L. (2025). Insight into volatile metabolites and key odorants in black teas processed from Jianghua Kucha tea germplasm (*Camellia sinensis* var. assamica cv. Jianghua). Food Chem..

[18] Zhu Y., Lv H.-P., Shao C.-Y., Kang S., Zhang Y., Guo L., Dai W.-D., Tan J.-F., Peng Q.-H., Lin Z. (2018). Identification of key odorants responsible for chestnut-like aroma quality of green teas. Food Res. Int..

[19] Wang H., Hua J., Jiang Y., Yang Y., Wang J., Yuan H. (2020). Influence of fixation methods on the chestnut-like aroma of green tea and dynamics of key aroma substances. Food Res. Int..

[20] Xie J., Wang L., Deng Y., Yuan H., Zhu J., Jiang Y., Yang Y. (2023). Characterization of the key odorants in floral aroma green tea based on GC-E-Nose, GC-IMS, GC-MS and aroma recombination and investigation of the dynamic changes and aroma formation during processing. Food Chem..

[21] Guo X., Schwab W., Ho C., Song C., Wan X. (2022). Characterization of the aroma profiles of oolong tea made from three tea cultivars by both GC–MS and GC-IMS. Food Chem..

[22] Ye J., Wang Y., Lin S., Hong L., Kang J., Chen Y., Li M., Jia Y., Jia X., Wu Z. (2023). Effect of processing on aroma intensity and odor characteristic of Shuixian (*Camellia sinensis*) tea. Food Chem. X.

[23] Pastoriza S., Mesías M., Cabrera C., Rufián-Henares J.A. (2017). Healthy properties of green and white teas: An update. Food Funct..

[24] Kochman J., Jakubczyk K., Antoniewicz J., Mruk H., Janda K. (2020). Health Benefits and Chemical Composition of Matcha Green Tea: A Review. Molecules.

[25] Liu N., Shen S., Huang L., Deng G., Wei Y., Ning J., Wang Y. (2023). Revelation of volatile contributions in green teas with different aroma types by GC–MS and GC–IMS. Food Res. Int..

[26] Jiang Y., Huang D., Lu C., Ye S., Li L., Li T., Liu X., Chen B., Guo J., Lu L. (2024). Shorten spreading duration enhance the quality of summer Meitan Cuiya tea. Food Chem. X.

[27] Feng Z., Li Y., Li M., Wang Y., Zhang L., Wan X., Yang X. (2019). Tea aroma formation from six model manufacturing processes. Food Chem..

[28] Liu Z., Yang C., Luo X., Hu B., Dong C. (2021). Research on the online rapid sensing method of moisture content in famous green tea spreading. J. Food Process. Eng..

[29] Jiang H., Li Y., He R., Tan J., Liu K., Chen Y., Liu H. (2022). Effect of Supplemental UV-A Intensity on Growth and Quality of Kale under Red and Blue Light. Int. J. Mol. Sci..

[30] He Y., Li J., Mei H., Zhuang J., Zhao Z., Jeyaraj A., Wang Y., Chen X., Li X., Liu Z. (2023). Effects of leaf-spreading on the volatile aroma components of green tea under red light of different intensities. Food Res. Int..

[31] Yu X., He C., Li Y., Zhou J., Chen Y., Yu Z., Wang Y., Ni D. (2021). Effects of different spreading treatments on the formation of aroma quality in green tea. Beverage Plant Res..

[32] Li J., Hua J., Dong C., Yang Y., Deng Y., Wang J., Jiang Y., Yuan H., Zhou Q. (2020). Real-time fingerprinting of the dynamics of green tea volatiles by ion mobility spectrometry for aroma assessment and discrimination. LWT.

[33] Ai Z., Zhang B., Chen Y., Yu Z., Chen H., Ni D. (2017). Impact of light irradiation on black tea quality during withering. J. Food. Sci. Technol..

[34] Chen Q., Zhu Y., Liu Y., Liu Y., Dong C., Lin Z., Teng J. (2022). Black tea aroma formation during the fermentation period. Food Chem..

[35] Zhang S., Chen L., Niu L., Yuan H., Shan X., Zhang Q., Feng Y., Zhou Q., Jiang Y., Li J. (2024). New insights into the role of lipids in aroma formation during black tea processing revealed by integrated lipidomics and volatolomics. Curr. Res. Food Sci..

[36] Wang Y., Zheng P., Liu P., Song X., Guo F., Li Y., Ni D., Jiang C. (2019). Novel insight into the role of withering process in characteristic flavor formation of teas using transcriptome analysis and metabolite profiling. Food Chem..

[37] Yang Z., Kinoshita T., Tanida A., Sayama H., Morita A., Watanabe N. (2009). Analysis of coumarin and its glycosidically bound precursor in Japanese green tea having sweet-herbaceous odour. Food Chem..

[38] Zhang Y., Lei P., Ding Y., Zhai X., Wan X., Li W., Zhang Y., Lv H.-P., Lin Z., Zhu Y. (2024). Uncovering characteristic and enantiomeric distribution of volatile components in Huangshan Maofeng and Zhejiang baked green teas. Food Chem..

[39] Wu H., Sheng C., Lu M., Ke H., Li T., Wei Y., Shen S., Yin X., Lu C., Wang Y. (2024). Identification of the causes of aroma differences in white tea under different withering methods by targeted metabolomics. Food Biosci.

[40] Chen M., Fang D., Gou H., Wang S., Yue W. (2022). Quantitative Measurement Reveals Dynamic Volatile Changes and Potential Biochemical Mechanisms during Green Tea Spreading Treatment. ACS Omega.

[41] Jumtee K., Komura H., Bamba T., Fukusaki E. (2011). Predication of Japanese green tea (Sen-cha) ranking by volatile profiling using gas chromatography mass spectrometry and multivariate analysis. J. Biosci. Bioeng..

[42] Zeng L., Zhou Y., Gui J., Fu X., Mei X., Zhen Y., Ye T., Du B., Dong F., Watanabe N. (2016). Formation of Volatile Tea Constituent Indole During the Oolong Tea Manufacturing Process. J. Agric. Food Chem..

[43] (2021). Methodology for Sensory Evaluation of Tea.

